# Silencing of a mannitol transport gene in *Phelipanche aegyptiaca* by the tobacco rattle virus system reduces the parasite germination on the host root

**DOI:** 10.1080/15592324.2022.2139115

**Published:** 2022-11-24

**Authors:** Vinay Kumar Bari, Dharmendra Singh, Jackline Abu Nassar, Radi Aly

**Affiliations:** a Department of Plant Pathology and Weed Sciences, Newe Yaar Research Station, Agricultural Research Organization (ARO), Ramat Yishay, Israel; b Department of Biochemistry, Central University of Punjab, Bathinda, India; c Department of Computational Sciences, Central University of Punjab, Bathinda, India

**Keywords:** Tobacco rattle virus, virus-induced gene silencing (VIGS), strigolactone, plant-parasitic weed, mannitol transport

## Abstract

Root parasitic weed *Phelipanche aegyptiaca* is an obligate plant parasite that causes severe damage to host crops. Agriculture crops mainly belong to the Brassicaceae, Leguminosae, Cruciferae, and Solanaceae plant families affected by this parasitic weed, leading to the devastating loss of crop yield and economic growth. This root-specific parasitic plant is not able to complete its life cycle without a suitable host and is dependent on the host plant for nutrient uptake and germination. Therefore, selected parasitic genes of *P. aegyptiaca* which were known to be upregulated upon interaction with the host were chosen. These genes are essential for parasitism, and reduced activity of these genes could affect host-parasitic interaction and provide resistance to the host against these parasitic weeds. To check and examine the role of these parasitic genes which can affect the development of host resistance, we silenced selected genes in the *P. aegyptiaca* using the tobacco rattle virus (TRV) based virus-induced gene silencing (VIGS) method. Our results demonstrated that the total number of *P. aegyptiaca* parasite tubercles attached to the root of the host plant *Nicotiana benthamiana* was substantially decreased in all the silenced plants. However, silencing of the *P. aegyptiaca MNT1* gene which encodes the mannitol transporter showed a significantly reduced number of germinated shoots and tubercles. Thus, our study indicates that the mannitol transport gene of *P. aegyptiaca* plays a crucial role in parasitic germination, and silencing of the *PaMNT1* gene abolishes the germination of parasites on the host roots.

## Introduction

The *P. aegyptiaca* is an obligate root-specific parasitic plant belonging to the Orobanchaceae family that represents a great challenge to economically important crops and the agriculture economy.^1,2^ Due to the lack of functional chloroplasts, these parasites are not able to synthesize organic solutes and depend on the host plant for their survival.^3^ The germination of this parasite weed is induced by a family of chemical molecules known as strigolactones (SLs) exuded into the rhizosphere by the host plant's root.^4,5^ After germination, a special kind of interconnection between host vascular tissue and the parasitic plant is established called the haustorium, and it is the main characteristic feature of parasitic plants.^6^

Haustorium helps parasites withdraw organic acids, sugars, ions, and amino acids from the host, potentially damaging the host crop and greatly reducing the yields in terms of quality and quantity.^7,8^ However, the nature of the solute molecules and their role in host–parasite interaction is less explored in *P. aegyptiaca*. Previous studies reported that during the initial phase of host-parasite attachment major alteration in metabolism occurs, and transported amino acids and sugars are two main organic solute molecules that are mainly altered in the parasite during the establishment of root connection with the host.^9^ Studies using primary metabolic profiling and compa-rative metabolomics of the early development of parasitic plants *P. aegyptiaca* and facultative hemiparasite *Triphysaria versicolor* before and after attachment to the hosts revealed that the levels of many specific metabolites increased after its attachment to the host root.^9^

Several organisms under stress conditions accumulate certain specific compounds in high concentrations that are still ‘compatible’ with their normal physiological processes, such as sugars, polyols, amino acid, or their derivatives.^10^ Sugar alcohols or polyols, such as mannitol is the most widely distributed soluble carbohydrate molecule that exists in biological systems.^11^ It can serve as a compatible solute and accumulate within the cell without disrupting cellular function. Mannitol has also known to play an important role in osmotic homeostasis by acting as an antioxidant.^12^ The existence of the mannitol cycle in *Orobanche* species suggests that possibly mannitol has an important physiological role in these parasites as well, which might be involved in osmotic homeostasis or protection against ROS species.^13,14^

Mannitol is synthesized in plants from mannose-6-phosphate through the action of an enzyme NADPH-mannose-6-phospho-reductase (M6PR).^15^ This enzyme catalyzes the conversion of mannose-6-phosphate to mannitol 1-phosphate, which is then dephosphorylated by a phosphatase to form mannitol.^16^ Mannitol acts as a transport form of sugar molecules synthesized in the leaves and translocated via the phloem to sink tissues in plants where they can be involved in the synthesis of fructose 6-phosphate and sucrose molecules.^17^

Previous studies in *Orobanche ramosa* identified the role of mannose 6-phosphate reductase (M6PR) in having a two-fold increase in the enzyme activity during tubercle enlargement and crown root formation with increased mannitol content in the attached parasite.^18^ Silencing of the parasite-specific M6PR gene in *Orobanche aegyptiaca* by siRNA resulted in decreased mannitol concentration in the haustorium tubercle and increased tubercle mortality, thus clarifying the role of mannitol in parasitism during the early phase of infection.^19^ A recent study further corroborated that transient silencing of *PaM6PR* in *P. aegyptiaca*, out of three genes (*PaM6PR, PaCWI*, and *PaSUS1*) involved in the osmotic regulation process causes the highest decrease in the total amount of reducing sugars than the control when grown on tomato host.^20^

Previous studies also showed that Methionine synthase from *Orobanche ramosa* (*Or-MET1*) was more prominently induced in tubercles and modulates the growth of *Orobanche*.^21^ In addition, the germination of *P. aegyptiaca* is highly sensitive to strigolactones (SLs), suggesting that *P. aegyptiaca* contains components of the SL signaling pathway, the *MAX2* (more axillary growth 2) gene is a component of the SL signaling pathway, which regulates a wide range of biological processes.^22^

A recent comparative transcriptomic study using differential expression (DE) analysis explored several genes which are essential to parasitism in *P. aegyptiaca*.^23^ These parasitic genes belonging to ATP-Binding Cassette (ABC) transporter such as pleiotropic drug resistance (PaPDR1) involved in SL transport,^24^ E3 ubiquitin ligase (PaUBQ3) a regulator in SL phytohormone signaling pathways,^25^ phosphate transporter (PaPHT1) involved in phosphate uptake from the soil,^26^ oligopeptide transporter (PaOPT1) involved in glutathione transport,^27^ glutamate receptor (PaGLR1) shown to be transcriptionally altered during in host–parasite interaction,^28^ sugar transporter (PaSUT1) involved in sucrose transport,^29^ CLP protease (PaCLP1) involved in protein degradation^30^ and amino acid transport families are coexpressed in parasitic stages and may be important in haustorial development and function. These candidate parasitism genes are involved in interaction with host plants and the transcript level of these genes was upregulated in response to haustorium-inducing factors before host attachment or during haustorial development following host attachment.^9,23^

In conclusion, these studies are highlighting the role of several genes that could be used to silence them in parasitic weeds *P. aegyptiaca* to achieve resistance in the host plant *N. benthamiana*. Based on the above research, we evaluated the effectiveness of the silencing of selected genes involved in host-parasitic interaction. To degrade the mRNA transcript of these genes, we used tobacco rattle virus-mediated gene silencing approaches^31^ in *P. aegyptiaca* and demonstrated that the mannitol transporter upon silencing significantly reduces the germination of *P. aegyptiaca* on the root of host plant *N. benthamiana*.

## Results

Based on the expression sequence tags data of the *P. aegyptiaca* deposited in the Parasitic Plant Genome Project (PPGP) database (http://ppgp.huck.psu.edu),^32^ we searched for and confirmed the existence of *P. aegyptiaca* specific gene(s) encoding for more axillary growth 2 (*PaMAX2-* regulators in SL signaling pathways GenBank: KX375414). Few *Orobanche* gene sequences were isolated from NCBI GenBank based on previous publications and a BLAST searched against *P. aegyptiaca* ESTs using the Parasitic Plant Genome Project to confirm the existence of those specific gene sequences in *P. aegyptiaca* such as mannitol transporter (*PaMNT1*) involved in polyols or mannitol transport, GenBank: AY136668, methionine synthase (*PaMET1*) involved in methionine biosynthesis GenBank: DQ849630. In addition, for other genes such as pleiotropic drug resistance (*PaPDR1*), sucrose transporter (*PaSUT1*), E3 ubiquitin ligase (*PaUBQ3*), oligopeptide transporter (*PaOPT1*), phosphate transporter (*PaPHT1*), CLP protease (*PaCLP1*), and glutamate receptor (*PaGLR1*) we performed a gene search in the *Arabidopsis thaliana* genome database and retrieved genes and it sequences were used as templates to perform a BLAST search against *P. aegyptiaca* ESTs from the Parasitic Plant Genome Project.^32^ Based on the above search, we have shortlisted selected genes (Table 1) and confirmed the suitable DNA sequences from non-homologous regions of these target genes that differ between *P. aegyptiaca*, and *N. benthamiana*, to avoid silencing of host genes **(Table S1)**.

**Table 1.** List of putative *P. aegyptiaca* genes used in this study

Candidate gene in *P. aegyptiaca* (as per PPGP database)	The notion of genes in the study	Insert size used for cloning in *pTRV2*	Best Arabidopsis Hit
>OrAe2FB1_3281: Length (2049 nt)	Mannitol transporter (PaMNT1)	275bp	AT3G18830
>OrAe2FB1_29: Length (2791 nt)	Methionine synthase (PaMET1)	231bp	AT3G03780
>OrAe2FB1_910: Length (1892 nt)	Sucrose transporter (PaSUT1)	268bp	AT1G22710
>OrAe42GB1_49526: Length (2821 nt)	Ubiquitin protein ligase (PaUBQ3)	213bp	AT5G05560
>OrAe3GB1_10228: Length (2451 nt)	Oligopeptide transporter (PaOPT1)	237bp	AT1G09930
>OrAe2FB1_52: Length (2119 nt)	Phosphate transporter (PaPHT1)	261bp	AT2G32830
>OrAe41G2B1_12298: Length (4604 nt)	ABC transporter (PaPDR1)	201bp	AT1G15210
>OrAe2FB1_82: Length (1918 nt)	ATP-dependent Clp protease (PaCLP1)	223bp	ATCG00670
>OrAe3GB1_77127: Length (1920 nt)	Glutamate receptor (PaGLR1)	202bp	AT2G24710
GenBank: KX375414	Maximum axillary growth 2 (PaMAX2)	215bp	AT2G42620

**Figure 1.**
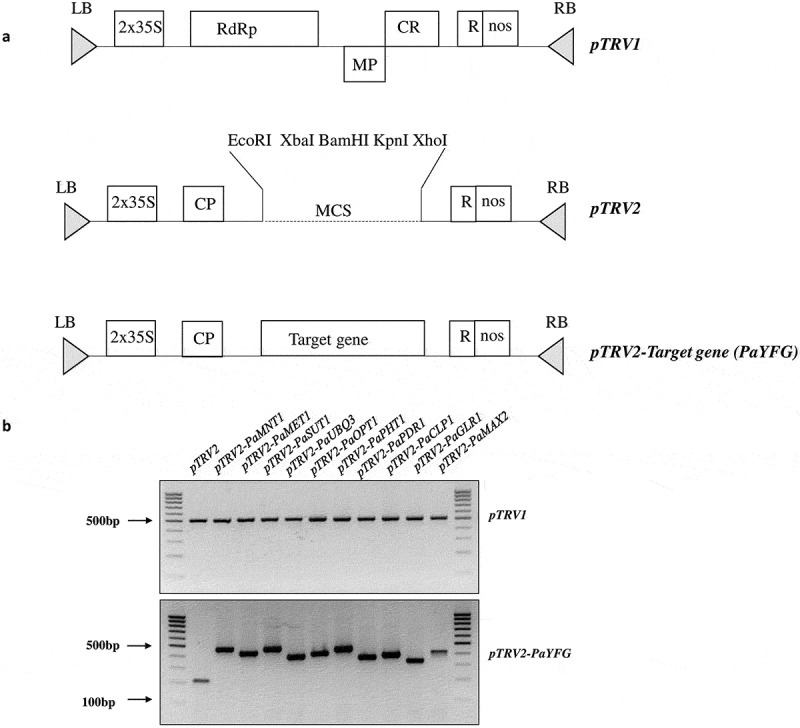
(a) Schematic representation of p*TRV1*, p*TRV2*, and p*TRV2-YFG* vector design used for the silencing *P. aegyptiaca* gene adapted from the previous study^35^. Abbreviations details -Left border (LB), a duplicated CaMV 35S promoter (2X 35S), 29KDa movement protein (MP), viral coat protein (CP), multiple cloning sites (MCS), Cystine rich protein (CR), 134 and 194 KDa replication protein (RdRp), self-cleaving ribozyme (R), nopaline synthase terminator (NOS-T), Right border (RB). (b) Expression of the empty plasmid *pTRV1* and *pTRV2* recombinant clones (*pTRV2-PaYFG*), in the VIGS-treated *N. benthamiana* plants. To confirm the infection PCR analysis of genomic DNA isolated from the leaves and roots was done 10–12 d after infection, both have given similar results.

**Figure 2.**
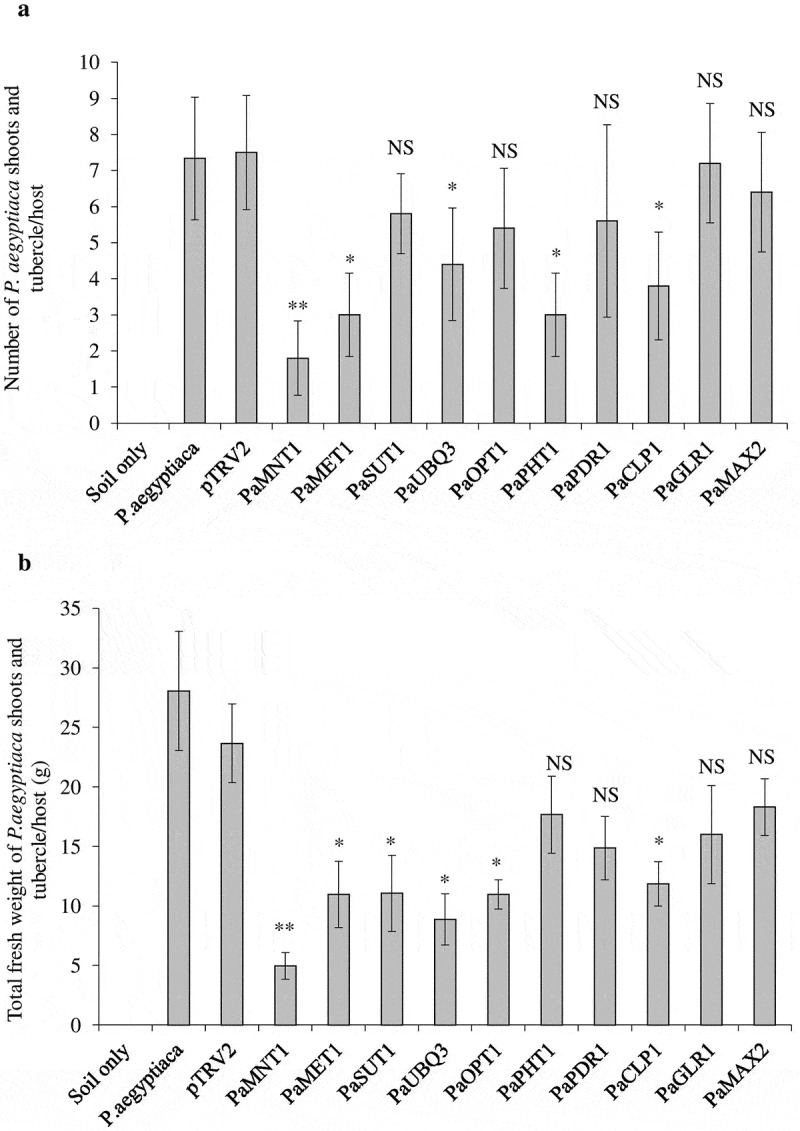
Reduced germination of *P. aegyptiaca* tubercles grown on host root *N. benthamiana*. The total number of *P. aegyptiaca* tubercles attached to the host plants in the pot assay. Tubercles that were larger than 2 mm (diameter) were counted and weighted. The number of total parasitic tubercles (a), and the total fresh weight of tubercles (b), were analyzed. Bars represent the average ±SE (n = 5) value from two different experiments with five independent host plants. Statistical differences were calculated with Student’s two-tailed t-test (p < .05). The asterisk on the bar indicates a significant difference between the *pTRV2*-mediated silenced line (*pTRV2-PaYFG*) compared to the vector control plants (*pTRV2*).

**Figure 3.**
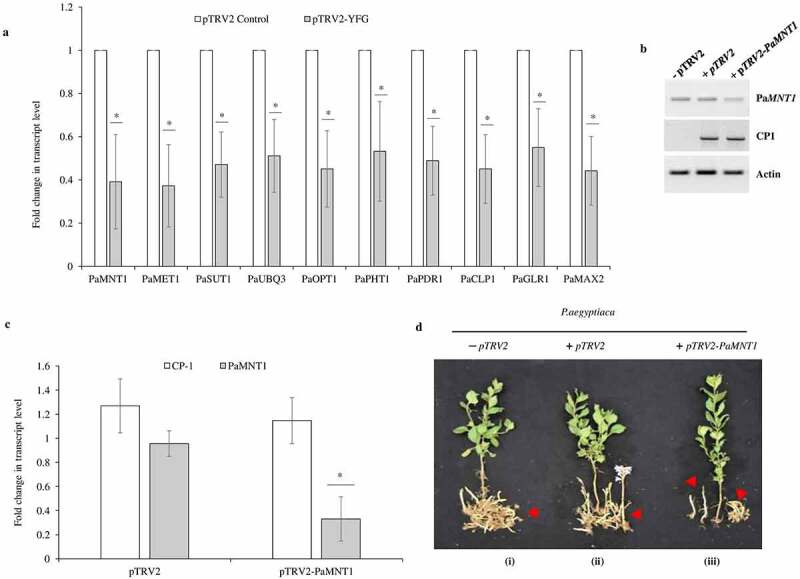
(a) The transcript level of various *pTRV2-PaYFG* silencing constructs using *P. aegyptiaca* tubercle. The expression level of each gene is displayed after normalization with the internal housekeeping gene *PaACT1*. All analyses were performed using three biological replicates. Data represented as average ± SD (n = 3). The asterisk on the bar indicates a significant difference between the *pTRV2-PaYFG*-silenced line compared to the vector control (*pTRV2*) plants as determined by JMP statistic software (P ≤ .05). (b, c) The transcript level of *PaMNT1*, viral CP-1 in *P. aegyptiaca* tubercle. Expression of viral coat protein transcript acts as a control to measure the infection level. The *P. aegyptiaca* actin gene served as an internal control for the expression. All analyses were performed using three biological replicates. Data represented as average ± SD (n = 3). The asterisk on the bar indicates a significant difference between the *PaMNT1*-silenced line compared to the vector control plants as determined by JMP statistic software (P ≤ .05). (d) Effect of the *P. aegyptiaca* target genes *PaMNT1*-silenced line compared to the vector control, on development of the parasite in *N. benthamiana* host plants. **(i)** host plant infected with *P. aegyptiaca* only **(ii)** host plant infected with *P. aegyptiaca* and agroinfiltrated with *pTRV1* and *pTRV2* vector **(iii)** host plant infected with *P. aegyptiaca* and agroinfiltrated with *pTRV1* and *pTRV2* vector containing fragment for silencing for *PaMNT1. P. aegyptiaca* tubercles and shoots attached to the controls and VIGS-treated *N. benthamiana* plants are indicated by red arrows.

To silence these selected genes of *P. aegyptiaca* (Table 1), we adopted the tobacco rattle virus (TRV) -based Virus-Induced Gene Silencing (VIGS) method.^33^ *pTRV* is a bipartite, positive-strand RNA virus with the *pTRV1* and *pTRV2* genomes. For virus-induced gene silencing (VIGS), only the *pTRV2* genome is genetically modified by introducing suitable gene-specific DNA fragments of the target gene (around 200–275 nucleotides for each gene) (Figure 1a) and delivered into the plant (along with the *pTRV1* genome) by agroinoculation.^34^ To avoid putative silencing of the host genes, non-homologous DNA sequences of the targeted gene(s), that differ between the host and the parasite, were chosen **(Table S1)**. The selected target regions of the individual genes from *P. aegyptiaca* were cloned in *pTRV2* using suitable restriction sites available at multiple cloning site (*BamHI/ XbaI/ EcoRI/ XhoI or KpnI*) which will not digest our selected region of interest. Recombinant clones were identified by diagnostic PCR using a primer outside the inserted DNA sequence in *pTRV2* and further confirmed by “Sangers” DNA sequencing **(Table S2)**. *pTRV2-PaYFG* (YFG-the desired gene sequences in *pTRV2* plasmid), and *pTRV1* were separately transformed in *Agrobacterium tumefaciens* strain EHA105, which is extensively used for plant transformation. Host plant *N. benthamiana* (5–6 leaves stage) were grown in the greenhouse and after 2 weeks, they were transferred to new pots containing *P. aegyptiaca* seeds (15 mg/kg soil). The host plants were agroinfiltrated with *A. tumefaciens* EHA105 containing the recombinant *pTRV2:PaYFG* or *pTRV2* and *pTRV1* both according to the method described previously.^33^ To confirm the *A. tumefaciens* EHA105 mediated transformation and accumulation of *pTRV1* and *pTRV2: PaYFG* in the *N. benthamiana*, genomic DNA from host plants leaves or roots were extracted using a plant genomic DNA extraction kit and subjected to diagnostic PCR to confirm virus infection after 10–12 d of agroinfiltration (Figure 1b). After 30 d of agroinfiltration, the host plant *N. benthamiana* was assayed for resistance to *P. aegyptiaca*, which was pre-infected with the parasite seeds (15 mg/kg soil) in the greenhouse. The total number of *P. aegyptiaca* tubercles and total fresh weights of the tubercles more than 2 mm was determined on *pTRV2:PaYFG* and *pTRV2* control plants. *pTRV2:PaYFG* treated plants expressing the target sequences of *PaMNT1, PaMET1, PaUBQ3, PaPHT1*, and *PaCLP1* cause a significant reduction in the total number of parasite tubercles grown on the host plant. In addition, the total fresh weight of tubercle, grown on host plant containing silencing vector against *PaMNT1, PaMET1, PaSUT1, PaUBQ3, PaOPT1, PaPHT1, PaPDR1, PaCLP1, PaGLR1*, and *PaMAX2* genes were found to be substantially decreased, as compared to the control plants treated with only *pTRV2* vector (Figure 2 a and b). To confirm the decrease in transcript level upon silencing of these genes, we analyzed the expression of all the genes of *P. aegyptiaca* which were silenced in the study using real-time PCR. Our results suggested that the expression of all the genes of *P. aegyptiaca* (*PaMNT1, PaMET1, PaSUT1, PaUBQ3, PaOPT1, PaPHT1, PaPDR1, PaCLP1, PaGLR1*, and *PaMAX2*) gets decrease on respective *pTRV2-PaYFG* infected host roots (Figure 3a). The transcript of viral coat protein was used as a measure to analyze the level of infection in the tubercles. Since the silencing effect was more effective in the case of *PaMNT1* to reduce the number and fresh weight of the tubercle, so we focused to analyzed the transcript level of the targeted *PaMNT1* grown on *N. benthamiana* host plants using quantitative real time-PCR. The expression analysis data showed that the transcript levels of *PaMNT1* were substantially reduced in the tubercles grown on host plants infected with recombinant *pTRV2-PaMNT1* as compared to only the *pTRV2* vector. Moreover, viral coat protein expression was similar in both *pTRV2* empty vector and *pTRV2-PaMNT1* treatments (Figures 3 b and c). We have also compared the morphological phenotype of the host plant grown on *PaMNT1* silenced condition which suggests very little to no growth defect in the host plant as compared to control without infection (Figure 3d). The silenced genes such as *PaUBQ3, PaPHT1*, and *PaCLP1* showed a reduction in the total number of parasitic tubercles grown on the host plant, and *PaSUT1, PaUBQ3, PaOPT1*, and *PaCLP1* showed a significant reduction in total fresh weight. Reduction in the total number of tubercles or fresh growth suggests that these genes could be important for the development of tubercles on host roots. Results from our study also suggested that out of all these genes mannitol transporter gene evolved as a significant contributor to shoots and tubercle growth on host roots. Since the mannitol transport gene plays an essential role in the shoots and tubercle growth hence disruption of the mannitol transporter gene could be used as a method to reduce the growth of parasitic weed *P. aegyptiaca* on host plants.

## Discussion

The root-parasitic weeds *Orobanche* and *Phelipanche spp*. mainly affect the roots of economically important crops throughout the semiarid regions of the world, especially the Mediterranean and the Middle East. These parasitic weeds are regarded as some of the most serious pests for the crops and completely depend on the host plant for their nutritional requirements.^36,37^ Control of these root-specific parasitic weeds, such as *Phelipanche* and *Orobanche spp*. is a challenging issue.^36^ Therefore, an innovative solution to the problem is urgently required.

Our major interest is focused on the identification of a specific gene out of all the genes we used in the study that upon silencing adversely affects the germination of *P. aegyptiaca*. In this article, we have tested the role of selected genes of the parasite *P. aegyptiaca* showing early upregulation after exposure to the host root and known to be involved in parasitism in particular in relation to metabolism and transport of sugars and amino acids (and oligopeptides) and major processes (proteolysis and transport of phosphate). Previous studies with *P. aegyptiaca CCD7* and *CCD8* genes of the strigolactone biosynthesis pathway suggested that silencing of these genes retarded the growth of parasites on the host.^38^ Using a similar silencing approach, we reduced the transcript level of the selected gene(s) involved in host–parasite interaction and demonstrated that the degree of infestation is strongly reduced for five silenced lines (*PaMNT1, PaMET1, PaUBQ3, PaPHT1*, and *PaCLP1*) and for the host plants *PaMNT1* resistance is correlated with the strong inhibition of the *MNT1* target gene expression in parasitic tubercle.

Previous studies described the existence of the mannitol cycle in the host root attached to *Orobanche ramosa* and *Orobanche crenata* a sister species to *P. aegyptiaca*.^13^ While considered to be synthesized in tubercle from the host-derived sucrose, mannitol and hexoses are mainly accumulated in shoots, before and after emergence, as already reported earlier.^13^ Mannitol was detected in broomrape before its attachment to the host root; however, their accumulation is strongly enhanced following the haustorial connection to the host root.^18^ This supports the occurrence of long-distance transport of these sugars in broomrape. The high mannitol concentrations are maintained in shoots during all the stages of parasitic plant development in *P. aegyptiaca* and *T. versicolor*, supporting the essential role of these sugars in the high sink strength for water and sucrose of the parasite.^9^ In that sense, silencing *PaMNT1* results in a decreased capacity to accumulate sugars from the host which affects the germination of *P. aegyptiaca*. Moreover, the tubercle is grown on a host plant with silenced genes such as *PaMET1, PaUBQ3, PaPHT1*, and *PaCLP1* there is less effect on total shoot number and tubercle fresh weight as compared to the *PaMNT1* treated plants. Hence, from this study, we concluded that a reduction in the transcript level of *PaMNT1*-mRNA in tubercles affects tubercle development (total number and fresh weight), which substantially affects the development of *Phelipanche* on the host root. Our study also concluded that out of all the parasitism genes used in the study, though they are playing important role in host–parasite interaction, silencing of *PaMNT1* could be used as a control strategy against these root-specific parasitic weeds.

## Material and methods

The complete sequences of *P. ramosa* mannitol transporter (GenBank: AY136668.1), methionine synthase (GenBank: DQ849630.1), and sucrose transporter (GenBank: KR559018.1) were selected from UniProt, and BLAST was performed against *P. aegyptiaca* using the PPGP database (http://ppgp.huck.psu.edu).^32^ For other candidate genes, the Arabidopsis genome database (TAIR)^39^ was chosen as a model, and the existence of homologous genes in *P. aegyptiaca* was confirmed using BLAST and reverse BLAST hosted by PPGP. The highly matched homologous sequences of respective candidate genes with the minimum e-value score were selected. In addition to designing the silencing construct using a target, sequence alignment was done with the host gene sequence using T-coffee and Clustal Omega software, and suitable mismatch regions in the *P. aegyptiaca* gene were selected for the VIGS **(Table S1).**

### *pTRV2* vector construction

The selected gene of *P. aegyptiaca* was aligned with the corresponding homolog in host *N. benthamiana* and the less conserved mismatch region was chosen as the selected region for the cloning in *pTRV2* to silence the gene in *P. aegyptiaca*. The region of interest (200–275nt) was amplified using the forward and reverse oligos flanking with restriction sites and cloned into a *pTRV2* vector using *BamH*I and *Xba*I or other suitable restriction sites **(Table S2)**. The recombinant clones containing the insert were confirmed with diagnostics PCR and Sanger DNA sequencing analysis and transformed in *Agrobacterium* strain EHA105. No modification was performed in the *pTRV1* vector in the study.

### Virus-induced gene silencing assay

Virus-induced gene silencing (VIGS) is a technology for the transient knock-down of target genes that are based on sequence-specific RNA degradation triggered by double-stranded RNA (dsRNA).^33^ Tobacco rattle virus-mediated gene-silencing was performed as described previously^33^ using the host *N. benthamiana* seedlings that had 5–6 leaves. In brief, the following plasmids: *pTRV1* and *pTRV2* or *pTRV2:PaYFG* (*pTRV2* carrying the *P. aegyptiaca* selected genes fragments) were separately transformed into *Agrobacterium tumefaciens* strain EHA105 using electroporation, and transformants were selected on rifampicin (20 µg/ml) and kanamycin (50 µg/ml) plate with 28°C incubation and positive transformants were confirmed by diagnostic PCR using *pTRV2* based oligos and Sangers DNA sequencing.

### Agroinfiltration

Agroinfilteration experiments were performed with *N. benthamiana* seedlings that had 5–6 leaves. Agroinfiltration of tobacco leaves was performed as described.^31^ In brief, the following plasmids: *pTRV-RNA1 (TRV), pTRV-RNA2*, and *pTRV-RNA2* with *P. aegyptiaca* silencing genes were transformed in *Agrobacterium* strain EHA105 using standard protocols. Single colonies were inoculated for primary broth culture (5 ml), followed by secondary broth culture (50 ml) in the presence of rifampicin and kanamycin antibiotics. The colonies were then grown overnight at 28°C. The next day, 50 ml of cell culture was pelleted by centrifugation at 3000 rpm for 15 min. The recovered pellet was dissolved in infiltration medium (10 mM MES; 10 mM MgCl_2_; 250 μM acetosyringone in double-deionized water) adjusted to an OD of 1.0 (600 _nm_), and then incubated at room temperature for 3 h. Just before infiltration, a culture of *pTRV1* and *pTRV2* in a 1:1 (v/v) ratio was prepared in infiltration buffer (0.0976 g of MES in 100 ml of water (5 mM), adjusting the pH-5.6). *Agrobacterium* mixture was introduced into the lower surface of the *N. benthamiana* leaf with a 2.0 ml syringe. Moreover, a booster dose was given after 1 week of the previous agroinfiltration. To confirm the infection in host plants, genomic DNA is extracted from host roots after 10 d and subjected to PCR using primer specific to *pTRV1* and *pTRV2.*

### Evaluation of host-plant resistance to the parasite

*P. aegyptiaca* seeds were collected from an infested tomato field in the Bet She’an Valley in eastern Israel. Host plant *N. benthamiana* was grown in the greenhouse with conditions of 24°C, 40%–80% moisture content, with alternating 12 hours of light and dark photoperiod. *N. benthamiana* plants were used as hosts for *TRV* infection. Host plants were germinated into 2–3 L pots filled with vermiculite culture soil (light-medium clay with 63% sand, 12% silt, and 22% clay).  . Grown seedlings of the host plant transferred to 5–10 L pots containing natural and vermiculite culture soil mixture were infested with *P. aegyptiaca* seeds (15 mg/kg soil). After 10 d, agroinfiltration was performed on the lower side of the host leaves. Host roots from *pTRV2*-VIGS and control plant treatments were collected 40–45 d after exposure to the *P. aegyptiaca* seeds and tubercles larger than 2 mm were counted, weighed, and RNA was then isolated from those tubercles for the analysis of target gene expression.

### RNA isolation and qRT‑PCR

Total RNA from parasite tubercles was extracted using a Spectrum plant total RNA kit (Sigma- STRN50-1KT) according to the manufacturer’s protocol. Total RNA (500 ng) was used to obtain cDNA according to the protocol of the Quanta Bioscience cDNA synthesis kit. The qRT-PCR analysis was performed in a volume of 10 μL using PerfeCTa SYBR Green FastMix, ROX (Quanta Biosciences), and 5X diluted cDNA as the template. *P. aegyptiaca ACT1* was used as the internal reference gene. The specificity of the primers was confirmed by melting curve analysis. The generated Ct values of the target genes were normalized using the reference gene *PaACT1*. Relative expression was calculated using the 2^−ΔΔCt^ method and expressed as a fold change concerning the control.^40^

## Supplementary Material

Supplemental Material

## Data Availability

All the data is provided as an electronic supplement. For any other relevant information available on request to the corresponding author.

## Authors’ information

Jackline Abu Nassar, Radi Aly: Department of Plant Pathology and Weed Research, Newe Ya’ar Research Center, Agricultural Research Organization (ARO), Volcani Center, Ramat Yishay, Israel.

Vinay Kumar Bari: Department of Biochemistry, School of Basic Sciences, Central University of Punjab, VPO-Ghudda, Bathinda, India.

Dharmendra Singh: Department of Computational Sciences, School of Basic Sciences, Central University of Punjab, VPO-Ghudda, Bathinda, India.
