# Supplementary material for: Silencing of a mannitol transport gene in Phelipanche aegyptiaca by the tobacco rattle virus system reduces the parasite germination on the host root
Source: Plant Signal Behav. 2022 Nov 24;17(1):2139115. doi: 10.1080/15592324.2022.2139115 (PMC9704376; doi:10.1080/15592324.2022.2139115)
Supplement: Supplemental Material [file KPSB_A_2139115_SM5407.pdf]

Supplementary information

Silencing of a mannitol transport gene in *Phelipanche aegyptiaca* by the tobacco rattle virus system reduces the parasite germination on the host root

Vinay Kumar Bari[1,2] *, Dharmendra Singh[3], Jackline Abu Nassar[1] & Radi Aly[1] *

*Correspondence

(Email: radi@volcani.agri.gov.il) https://orcid.org/0000-0002-6038-9586

(Email:vinay.bari@cup.edu.in) https://orcid.org/0000-0002-5670-3798

Table S1.

Sanger DNA sequencing of each silenced construct. The red color highlighted was used in the cloning of the fragments in the pTRV2 vector.

**1**	**TRV2-PaMNT1**
	NNNNNNNNGNNNNNNNNNAGCNCGNNGAGCTTTATTATTACGGNNGANNGGNCTTAGANTCTGTGAGTAAGGTTACCGAATTCTCTA<br>GAAGTATGCTTTCGCTTGTTCTTTCTTGGCCTCCATGACCTCCATCTTACTTGGATATGATATCGGAGTGATGAGTGGAGCCATAATAT<br>ACATCGAGAAATCGATCAAGATGACTTACGTTCAAAAGGAGGTCATAATGGGAATCTTAAATCTCTACTCGCTCATCGGGTCGGCTG<br>CCGCCGGCCGGACCTCCGATTGGATCGGACGCCGGTACACCATAATCTTCGCCGGANCCATTTTCTTTGTCGGTGCTTTGCTGATGGG<br>TTTCGCGACTAANGATCCGGTACCGAGCTCACGCGTCTCGAGGCCCGGGCATGTCCCGANNACATTAAACTACN
**2**	**TRV2-PaMET1**
	NNNNNNNNTGNNNNNNNNNAGCNCGNNGAGCTTTATTATTACGGACNANNGNNCTTAGANTCTGTGAGTAAGGTTACCGAATTCTCT<br>AGATGTCACATACTTGCTGCTTTCTAAACCCGCTAAGGGTGTTGAGAAAACTTTTCCTCTTCTTTCACTTCTTGACAAAATCCTCCCAA<br>TCTACAAGGAAGTTATTGCTGAGCTGAAGGCAGCAGGTGCTTCATGGATCCAGTTTGATGAGCCCACCCTAGTTCTGGATCTCGAGT<br>CTCACCAGCTGGAAGCATTCACCAAGGCTTATGCTGAACTCGAGTCATCTTTGTCTGGGGATCCGGTACCGAGCTCACGCGTCTCGA<br>GGCCCGGGCATGTCCCGAANACATTAAACTACGGTTCTTTAAGTAGATCCGTGTCTGAAGTTTTAG
**3**	**TRV2-PaSUT1**
	NNNNNNNNNGNNNNNNNNNNNNNNNCGNNGNGCTTTATNATTACGGACGAGTGGNCTTAGANTCTGNGAGTAAGGTTACCGAATTCTC<br>TAGAACTCCGTACGTGCAGTTGCTGGGCATACCGCACAAATGGGCCGCTTTTATCTGGCTGTGCGGCCCGATTTCGGGATTGCTGGTC<br>CAGCCCATAGTCGGGTTCTACAGCGATAATTGCACCTCANGGTTCGGACGGCGGCGCCCCTTCATCGCCGCCGGCGCGGGGCTCGTG<br>GCGGTGGCGGTTTTCCTGATCGGGTTCGCCGCCGATTTGGGGCATGCCGGCGGCGATTCGCTCGAGAAGGCTGCGAAGCCTAGGGCT<br>ATTGCTGTGTGGATCCGGTACCGANCTCACGCGTCTNGANNCCCGGGCATGTCCCGAANACATTAAACTACNGNTCNTTTNAGNTAN<br>AT
**4**	**TRV2-PaUBQ3**

	NNNNNNNNNNNNNNNNNNNNNNCGNNGAGCTTTATTATTACGGACGAGTGGNCTTAGATTCTGTGAGTAAGGTTACCGAATTCTCC<br>TTGCAGCGTGGATGTGGTTCTGCCATCATTAGTGATCTTCCACCTCTGATCTGCAAAGATGGAATTTCTGTTGCGAATTGGGCCCGCA<br>AGANTGTTGCTTTTTATAGTCTGTTGTGCGGTGCAGACCTATCAGGAAATAGGCTCTCGTCAGGTGTTACTTGCAGTATTGCAGCTGG<br>ATTATATCATACTCGGGAAGAGCTTACAGTTTTGGGCTCGAGGCCCGGGCATGTCCCGAANACATTAAACTACGGTTCTTTAAGTAG<br>ATCCGTGTCTGAGTTTTAGA
**5**	**TRV2-PaOPT1**
	AGNNNNNNNGTNNCNNNNNNNNNCGNNGNNCTTTATTATTACGGNCGAGTGGNCTTAGNNTCTGTGAGTAAGGTTACCGAATTCTC<br>TAGAGCTAAGTTTTTCCTTATTGCACTAGTCTGCAGTTTTTCCTGGTACCTACTTCCAGGTTACCTCTTCCCAACTCTCACTAGCATTTC<br>ATGGATTTGTTGGACATTCTCAAAGTCTGTCACGGCACAACANNTTGGATCGGGCCTAANANGCCTGGGCCTTGGAGCGTTAACATT<br>AGATTGGACTGCTGTGGCATCGTTCTTGTTCAGCCCACTCATATGTCCCTTCTTTGCCATAATGCTCGAGGCCCGGGCATGTCCCGAN<br>NNCATTAAACTACGGTTCTTTAAGTAGATCCGTGTCTGAAGTTTTAG
**6**	**TRV2-PaPHT1**
	NNNNNNNNNGNNNNNNNNANCNCGNNGAGCTTTATTATTACGGACGAGTGGACTTAGATTCTGTGAGTAAGGTTACCGAATTCTCT<br>AGAGCCCGTTTGGCATTCTTGGCAACCAACGCCGTGTACCGTGCCGTTTCGGGCATTTTCATACGCCAGTAGTAAGTCAGGGTCGCA<br>GGGAGGGCACCAAACATCAGGATAAGACGCCAAGCGTAATCGGCCTGAGGGATAGTCGAAAGAAGGGCATCTTCTGCATAAGAAG<br>GGGCGGGATATGCGGCCTTAAACGCGGCCGAGACTATAATCGCCACCATCCCACCGGCCAAAATCCCGAAACCCTGCATCGCGAAA<br>ACAGCNNNGNNNCGCGAAAACNNNNGNATC
**7**	**TRV2-PaPDR1**

	ANNNNNNNNGNNNNNNNNNAGCNCGNNGAGCTTTATTATTACGGACGANNGGNCTTAGANTCTGTGAGTAAGGTTACCGAATTCTCACGGGGAATATCAGGCGGACAGAAAAAGCGCCTCACGACTGGTGAAATGCTGGTTGGACCTGCAAAAGTTTTCTACATGGACGAAATCTCAACGGGTCTTGACAGTTCAACTACATTTCAAATTATTAAGTACATGAGGCAGATGGTTCATATCATGGATGTGACAATGATAATATCCCTTCTCCAACCGGCACCAGAAACCTCGAGGCCCGGGCATGTCCCGAANACATTAAACTACGGTTCTTTAAGTAGATCCGTGTCTGAAGTTTTAGA
**8**	**TRV2-PaCLP1**
	NNNNNNNNNNGNNNNNNNNNNAGCNCGANGAGCTTTATTATTACGGACGNNNNGGACTTAGANTCTGTGAGTAAGGTTACCGAATTCTCGTCCTGGCGGGTCAGCAATGTATGCATTAGCTATTTATAATATGATGCAATTTATAAACCCAGATGTGAATACAATAGGACTAGGAATAGCCGCATCAATGGGATCTATGATCCTGGCTGGGGGAGCAATTCGTATAGCATTACCTCACTTAAGAGTAATGATCCGTCAACCTGTATGGGGTTATTCTGGGTGGATTAGTGCAAGCGATTGTGTACGGAGAGCTCGAGGCCCGGGCATGTCCCGAANACATTAAACTACGGTTCTTTAAGTAGATCCGTGTCTGAAGTTNNN
**9**	**TRV2-PaGLR1**
	GGCGACGGACGACCAAATCTCCACCGAGCTTTACAAGCTGATGACTATGCAAACACGAGTTTTCGTGGTCCACATGTTGGGTCCACTTGCTTCCCGCCTTTTTACCAAAGCGAAACAGTTAGGAATGATGAGTCGGGAGTATGCATGGATAATAACGGACGGCATCACGAACGAACTAAACTCGATGGACCGTTCGGTTATC
**10**	**TRV2-PaMAX2**
	ACCCGTTACTCACTCACCCTCCGGGGCAACCTCCGCGACATCTTCATGGTCCCCACCTGCTTCCAGTCCATATCCCACCTCGACCTCTCTGTCCTCTCCCCGTGGGGCCACCCTCTCACCTCCGCCGACGATCCCGACTCCGAACTCGTCGGCCACGTCCTCGGCCAAGCCTTCCCGTCCGTCACCTCCCTCTCGCTCTATGCCCGTAACCCTTC

Table S2. List of Oligos used in the study

Oligos name	Sequence 5’-3’ (target sequences are shown in red and RE sites are underlined)	Use in the study
TRV1-DG-F	CGTGCTAATTGGATTTTGG	Validation of transformation
TRV1-DG-R	TGCAGAGCAGGAACTCTATC	
TRV2-SEQ-F	GCGGTTCTTGTGTGTCAAC	Validation of transformation
TRV2-SEQ-R	CTAAAACTTCAGACACGGATCTAC	
PaMNT1-Xba2F	GAGTAAGGTTACCGAATTCTCTAGAAGTATGCTTTCGCTTGTTCTTTC	Cloning *P. aegyptiaca* mannitol transporter fragment in TRV2 vector-Blast hit only Orobanche, target region +275bp
PaMNT1-BamH2R	GACGCGTGAGCTCGGTACCGGATCCTTAGTCGCGAAACCCATC	
PaMET1-Xba1F	ACCGAATTCTCTAGATGTCACATACTTGCTGCTTTC	Cloning *P. aegyptiaca* Methionine synthase fragment in TRV2 vector-Blast hit only Orobanche target region +231bp,
PaMET1-Kpn1R	TCGGTACCGGATCCCCAGACAAAGATGACTCGAG	

PaSUT1-Xba1F	ACCGAATTCTCTAGA*ACTCCGTACGTGCAGTTG*	Cloning *P. aegyptiaca* Sucrose transporter 1 fragment in TRV2 vector-Blast hit only Orobanche target region +268bp
PaSUT1-BamH1R	TCGGTACCGGATCC*ACACAGCAATAGCCCTAGGC*	
PaMAX2-Xba1F	GAGTAAGGTTACCGAATTCTCTAGA*ACCCGTTACTC ACTCACCC*	Cloning *P. aegyptiaca* MAX2 fragment in TRV2 vector-Blast hit only Orobanche target region +215bp
PaMAX2-BamH1R	GACGCGTGAGCTCGGTACCGGATCC*GAAGGGTTACG GGCATAG*	
OPT1-XbaI-F	GGACTTAGATTCTGTGAGTAAGGTTACCGAATTCTC TAGAGCTAAGTTTTTCCTTATTGCAC	Cloning *P. aegyptiaca* oligopeptide transporter OPT4 fragment in TRV2 vector-Blast hit only *Orobanche* target region +237bp
OPT1-XhoI-R	TTTAATGTCTTCGGGACATGCCCGGGCCTCGAGCAT TATGGCAAAGAAGGGAC	
PaPHT1-Xba1-F	GAGTAAGGTTACCGAATTCTCTAGAGCCCGTTTGG CATTCTTGGC	Cloning *P. aegyptiaca* Phosphate transporter fragment in TRV2 vector-Blast hit only *Orobanche* target region +261bp
PaPHT1-BamH1-R	GACGCGTGAGCTCGGTACCGGATCCGCTGTTTTCGC GATGCAGGG	

PaGLR1-EcoRI-F	CGGACGAGTGGACTTAGATTCTGTGAGTAAGGTTACCGAATTCTCGGCGACGGACGACCAAATC	Cloning *P. aegyptiaca* Glutamate receptor fragment in TRV2 vector-Blast hit only Orobanche target region +202bp
PaGLR1-XhoI-R	TAGTTTAATGTCTTCGGGACATGCCCGGGCCTCGAGGATAACCGAACGGTCCATCGAG	
PaCLP-EcoRI-F	CGGACGAGTGGACTTAGATTCTGTGAGTAAGGTTACCGAATTCTCGTCCTGGCGGGTCAGCAATG	Cloning *P. aegyptiaca* CLP ATPase fragment in TRV2 vector-Blast hit only Orobanche target region +223bp
PaCLP-XhoI-R	TAGTTTAATGTCTTCGGGACATGCCCGGGCCTCGAGCTCTCCGTACACAATCGCTTGCAC	
PaPDR1-EcoRI-F	CGGACGAGTGGACTTAGATTCTGTGAGTAAGGTTACCGAATTCTCACGGGGAATATCAGGCGGAC	Cloning *P. aegyptiaca* ABC transporter fragment in TRV2 vector-Blast hit only Orobanche target region +201bp
PaPDR1-XhoI-R	TAGTTTAATGTCTTCGGGACATGCCCGGGCCTCGAGGTTTCTGGTGCCGGTTGGAG	
PaUBQ-EcoRI-F	GGACGAGTGGACTTAGATTCTGTGAGTAAGGTTACCGAATTCTCCTTGCAGCGTGGATGTGGTTC	Cloning *P. aegyptiaca* E3 Ubiquitin ligase fragment in TRV2 vector-Blast hit only Orobanche target region +213bp
PaUBQ-XhoI-R	TAGTTTAATGTCTTCGGGACATGCCCGGGCCTCGAGCCCAAAACTGTAAGCTCTTCCCGAG	
TRV1-RT-F	TTACAGGTTATTTGGGCTAG	qRT-PCR Analysis

TRV1-RT-R	CCGGGTTCAATTCCTTATC	
TRV2-CP-S1	CTGGGTTACTAGCGGCACTGAATA	
TRV2-CP-A1	TCCACCAAACTTAATCCCGAATAC	
PaMNT1-RT-F	CGACGAAAGCACTCATCAAT	
PaMNT1-RT-R	CCCGGTATCATATCCGAGAAGG	
MNT1-RT-F	GTACAGCCCGGAGATATTCAAG	
MNT1-RT-R	GATCCTGTCGAGTAAGAACGTG	
PaACT1-RT-F	TGGCATCACACGTTTTAC	
PaACT1-RT-R	AGCCTGGATAGCAACATAC	
